# Fever of unknown origin and splenomegaly in an immunosuppressed Swiss patient: case report of a Neoehrlichia mikurensis infection in a changing climate and review of the literature

**DOI:** 10.1099/acmi.0.001168.v3

**Published:** 2026-09-15

**Authors:** R. Martischang, C. P. Buclin, R. Bonnet, N. Bürgisser, O. Opota, A. Huttner, G. Greub

**Affiliations:** 1 Infectious Diseases Division, Geneva University Hospitals, Geneva, Switzerland; 2 Internal Medicine Division, Geneva University Hospitals, Geneva, Switzerland; 3 Service of Medical Diagnostic Microbiology, Lausanne University Hospital, Lausanne, Switzerland; 4 Center for Research on Intracellular Bacteria (CRIB), Institute of Microbiology, University of Lausanne, Lausanne, Switzerland; 5 Service of Infectious Diseases, Lausanne University Hospital, Lausanne, Switzerland

**Keywords:** fever of unknown origin, intracellular bacteria, *Neoehrlichia mikurensis*, pancytopenia, tick-borne infection, zoonotic infection

## Abstract

*Neoehrlichia mikurensis* is an emerging tick-borne pathogen that is increasingly reported and can cause fever of unknown origin in immunocompromised individuals. We report a case of neoehrlichiosis occurring after B-cell-depleting treatment. Despite the availability of effective and inexpensive treatment, patients often experience significant care delays due to diagnostic challenges and insufficient awareness.

## Data Summary

All data associated with this work are reported within the article.

## Introduction

*Neoehrlichia mikurensis*, a Gram-negative obligate intracellular bacterium (family *Anaplasmataceae*), is an emerging tick-borne pathogen. First detected in 1960 in a tick collection [1], it was only identified as a human pathogen in 2010 in Sweden and Switzerland [2, 3]. Since then, it has been increasingly reported across Europe and Asia [4].

This pathogen is transmitted by *Ixodes ricinus* in western Europe and *Ixodes persulcatus* in eastern Europe and Asia. In Europe, the prevalence of tick-borne infections is rising due to greater human exposure during outdoor activities and expansion of tick habitats associated with climate change. In Switzerland, the proportion of territory colonized by ticks increased from 16% in 2009 to 25% in 2019, largely driven by rising temperatures [5]. A parallel increase in *N. mikurensis* prevalence has also been documented in neighbouring Alsace, France, with an estimated *N. mikurensis* prevalence among ticks of 5.42% between 2013 and 2020, and an increasing trend over the years [6]. In Switzerland, a similar prevalence of *Neoehrlichia* (5.7%) was documented on ticks by PCR [7].

Neoehrlichiosis generally presents with nonspecific symptoms such as fever, malaise, night sweats, weight loss, fatigue and polyarthralgia. Skin rash (resembling erysipelas or erythema nodosum) and hepatosplenomegaly have also been reported. Complications may include vascular or thromboembolic events, liver cytolysis, cholestasis and cytopenia [4], ranging from isolated leucopenia or thrombocytopenia to bicytopenia and pancytopenia. Additionally, *N. mikurensis* has been associated with arterial aneurysms [4]. Co-infection between *N. mikurensis* and *Borrelia burgdorferi* has been reported and should be further investigated [4, 8]. Such co-infections might modify the pathophysiology of each pathogenic agent and their infectivity, potentially explaining unusual clinical presentations misattributed to *Borrelia*.

We aim to summarize the epidemiological characteristics, diagnostic and treatment strategy of neoehrlichiosis based on a case of fever of unknown origin (FUO). The patient provided written informed consent for publication of clinical data. This case report was prepared in accordance with the CAse REport guidelines [9].

## Case presentation

A 50-year-old woman was hospitalized in December 2024 in the internal medicine department of a Swiss tertiary hospital for FUO. Her history included multiple sclerosis treated with rituximab, which was discontinued in 2022 due to secondary immunoglobulin deficiency. She experienced persisting lymphopenia (CD4, CD56+CD16+ NK and CD19+) and pan-hypogammaglobulinaemia; complement levels were normal (Table 1). History also included immune-mediated diabetes of the adult, polyarthralgia of unclear aetiology since 2021 and chronic cholestasis, previously investigated by a liver biopsy in 2022, demonstrating numerous biliary microhamartomas. Three weeks prior to the current hospitalization, she experienced a COVID-19 infection. Two years earlier, she was hospitalized twice for a *Campylobacter* bacteraemia (in July 2022 and March 2023).

**Table 1.** Diagnostic strategy and management

Category	Investigations	Results (reference range)
**Laboratory values**	Complete blood count	Pancytopenia with lymphopenia 0.36 g l^−1^ (1.0–4.5), thrombocytopenia 90 g l^−1^ (150–350), anaemia 65 g l^−1^ (120–160)
	Bone marrow biopsy and haematological workup	No evidence of haematological malignancy, haemophagocytosis or intracytoplasmic morulae
	C-reactive protein (CRP)	15–50 mg l^−1^ (<10)
	Ferritin	252 µg l^−1^ (7–171)
	Procalcitonin	0.48 µg l^−1^ (<0.50)
	Creatinine	68 µmol l^−1^ (44–80)
	Alanine aminotransferase	19 U l^−1^ (9–42)
	Gamma-glutamyltranspeptidase	69 U l^−1^ (9–35)
	IgA	0.31 g l^−1^ (0.70–4.00)
	IgM	<0.05 g l^−1^ (0.40–2.30)
	IgG	5.44 g l^−1^ (7.00–16.00)
	IgG1 subclass	3.1 g l^−1^ (4.1–10.1)
	IgG3 subclass	0.1 g l^−1^ (0.1–0.8)
	Complement levels	Within normal limits (C3 and C4)
	Lymphocyte subsets (from 24/12/2024 to 22/04/2025)	CD3+ T lymphocytes: 673 → 984 cells/µl (690–2,540); CD56+/CD16+ NK cells: 3 → 42 cells/µl (50–590); CD19+ B lymphocytes: 1 → 133 cells/µl (90–660)
**Imaging**	Thoraco-abdominal computed tomography and Fluorodeoxyglucose Positron Emission Tomography-Computed Tomography (PET-CT)	Splenic infarcts without pathological hypermetabolism or lymphadenopathy
	Transthoracic echocardiography	No significant abnormality
	Fundoscopic examination	No retinal or ocular abnormalities
**Viral investigation**	Severe Acute Respiratory Syndrome Coronavirus 2 Reverse Transcription-Polymerase Chain Reaction	Positive at admission; negative after 2 weeks
	Epstein-Barr virus, Cytomegalovirus, parvovirus B19	Negative by serology and PCR
	Hepatitis A Virus, Hepatitis B Virus, Hepatitis C Virus serology	Negative
	HIV screening	Negative
**Bacterial/zoonotic investigation**	Blood cultures	Negative
	Interferon-γ release assay (IGRA)	Negative
	Serology for *Bartonella* spp., *Coxiella burnetii*, *Brucella* spp*.*, *Rickettsia* spp*.*, *B. burgdorferi*	Negative
	16S rRNA eubacterial PCR	Positive for N. mikurensis*
	Real-time TaqMan *N. mikurensis* PCR	Positive (Ct 21.6)
**Treatment**	Antimicrobial therapy	Doxycycline 100 mg twice daily for 28 days
**Follow-up/outcome**	Clinical response	Complete resolution of fever, fatigue, gastrointestinal symptoms, weight loss, polyarthralgia and pancytopenia
	Molecular response (Follow-up *N. mikurensis* PCR)	Day 4: Ct 29.2 Day 18–28: negative
	Three-month follow-up	Sustained clinical remission, negative PCR, normalization of cytopenias and lymphocyte subsets

* By amplicon sequencing.

The patient presented with fatigue, fever (38–38.8 °C), night sweats, weight loss and gastrointestinal symptoms from August 2024 onwards, including nausea, intermittent non-localized abdominal cramps, self-resolving mucous diarrhoea and ageusia. The polyarthralgia transiently improved after treatment with prednisone 20 mg daily but then relapsed. No neurological symptoms were reported.

The patient travelled to northern Italy (Lake Maggiore region) in 2020, where she had mosquito bites and occasionally hiked but detected no tick bites. She had regular contact with domestic cats and dogs but no exposure to farm animals or wildlife. She had no history of blood transfusions.

Laboratory findings showed pancytopenia and mildly elevated inflammatory markers (Table 1). The diagnostic approach in this patient with anti-CD20-associated immunodeficiency and FUO was ultimately guided by standard recommendations, as outlined in recent literature [10] and summarized in Table 1. This workup, including an additional bone marrow biopsy, was non-contributory, except for splenic infarcts on the PET-CT.

Given the presence of hypogammaglobulinaemia and lymphopenia, targeted viral investigations, including EBV, CMV, parvovirus B19, human immunodeficiency virus, viral hepatitis and SARS-CoV-2, were non-contributory (Table 1). Screening for HHV-6 and HHV-7 was not performed, given the absence of suggestive clinical features and the relatively moderate degree of immunosuppression.

Given the patient’s immunosuppression and exposure history (travel to Italy, cat contact and possible tick exposure), investigations for vector-borne and intracellular zoonotic pathogens were initially negative (Table 1). *Anaplasma* spp., *Ehrlichia* spp. and *Neoehrlichia* spp. were investigated at a later stage, upon our request, using a 16S rRNA broad-range PCR performed at CHUV in Lausanne (Switzerland).

Tuberculosis was screened using an interferon-γ release assay (IGRA). However, its sensitivity is reduced in immunocompromised patients, and it does not exclude active disease. In the absence of relevant exposure, respiratory symptoms and with a non-suggestive FDG PET-CT, the likelihood of tuberculosis was considered low. Investigations for non-infectious causes, including rheumatologic, endocrine and drug-related aetiologies, were conducted in parallel (Rheumatoid factor, Antinuclear Antibody, and Thyroid-Stimulating Hormone). The patient was discharged with planned outpatient follow-up.

Shortly after discharge, the broad-range 16S rRNA PCR was positive, and amplicon sequencing identified *N. mikurensis*. A species-specific *N. mikurensis* quantitative PCR confirmed this result (Ct of 21.6). Treatment with doxycycline 100 mg bid was initiated. Clinical, biological and viral clearance documented at day 28 (Table 1) supported discontinuation of her treatment, after a minimum treatment duration of 21 days. A reassessment at 3 months post-treatment confirmed bacteriological clearance and sustained remission. Notably, the patient’s T and B lymphopenia, initially attributed to anti-CD20 therapy, shortly resolved after treatment completion (Table 1).

## Discussion

Until 2023, only 16 human *N. mikurensis* infections have been reported in Switzerland [11]. The patient’s clinical presentation aligns with previously reported cases in the literature, including case reports and case series [4, 8, 12–14], presenting mainly with FUO, polymyalgia, pancytopenia, elevated inflammatory markers, mild liver test abnormalities and hepatosplenomegaly.

Human infections are mainly documented among immunocompromised individuals, with splenectomy, B-lymphocyte-depleting therapy, autoimmune disease or haematologic neoplasms [15]. In Norway, *N. mikurensis* seroprevalence was detected in 7.4% of 163 immunocompromised patients compared to 1.2% of 85 immunocompetent patients [16]. Specifically, B-lymphocyte-depleting therapies have been associated with *N. mikurensis* infection, likely due to impaired antibody production, reduced cytokine secretion and the disruption of B-lymphocyte antigen presentation to T lymphocytes [4, 12]. Among patients with multiple sclerosis, a recent case series observed an association between infection risk and B-cell depletion (CD19/20) but not with hypogammaglobulinaemia [14]. In this case, the patient’s lymphopenia likely contributed to persistent symptoms and the inability to control the *N. mikurensis* infection.

*N. mikurensis* is believed to target polymorphonuclear neutrophils and endothelial cells. Morulae are observed in infections caused by *Anaplasma phagocytophilum* and *Ehrlichia chaffeensis*, though never described with *N. mikurensis*. The presumed reservoirs of *N. mikurensis* include the liver, spleen, bone marrow and possibly the skin. Hepatosplenomegaly is frequently observed, likely due to splenic sinusoid and intrasinusoidal hepatic T-cell infiltration [4, 8]. Pancytopenia is occasionally reported, with *N. mikurensis* linked to bone marrow T-lymphocyte infiltration and considered a potential trigger for haemophagocytic lymphohistiocytosis [8]. The rapid resolution of both T- and B-cell lymphopenia shortly after treatment suggests that *Neoehrlichia* may have contributed to transient bone marrow dysfunction and impaired immune control. However, in the absence of additional testing (e.g. eubacterial PCR on the bone marrow biopsy), the underlying mechanisms remain uncertain.

The patient was asymptomatic from her travel in Italy (2020) until 2024, possibly due to a persistent infection. Persistent asymptomatic infection reactivating under B-cell-depleting treatment has been described in a population with B-cell lymphoma, exhibiting prior *N. mikurensis-*specific T-cell response at treatment initiation [13]. This prompts the need for an exhaustive history when determining the epidemiological exposure.

In the present case, the diagnosis was only confirmed after 5 months. The median diagnostic delay for this pathogen has been estimated at 60 days in a report of 11 cases [17]. In the absence of a clinical emergency, we recommend considering *Anaplasma* spp. and *Neoehrlichia* spp. as part of a second-line investigation for non-neutropenic anti-CD20-associated immunodeficiency FUO, even in the absence of a tick bite history.

Because *N. mikurensis* is an obligate intracellular bacterium and cannot be cultured on axenic media, diagnosis relies mainly on molecular methods, which demonstrate very good performance on blood, particularly given high bacterial load and lack of sensitivity of traditional culturing methods, and they should be considered first-line [18]. Available assays include the pan-bacterial 16S rRNA PCR, nested PCR targeting all members of the *Anaplasmataceae* family and *N. mikurensis*-specific PCRs targeting the 16S rRNA gene or *gro*EL genes [4, 8, 18].

At the Swiss National Reference Center for Tick-Borne Infections (CHUV, Lausanne, Switzerland), a broad-range eubacterial 16S rRNA PCR is performed on blood to detect members of the *Anaplasmataceae* family, including *Ehrlichia*, *Anaplasma* and *Neoehrlichia* species. Positive amplicons are subsequently subjected to Sanger sequencing and compared with public databases (e.g. NCBI) for species identification. In parallel, the CHUV laboratory also performs two real-time TaqMan quantitative PCRs targeting specifically *A. phagocytophilum* and *N. mikurensis*. These TaqMan PCRs are performed using an automated platform that combines (i) Magnapure 96-well DNA extraction robots (Roche, Switzerland), (ii) Applied Biosystems PCR machines and (iii) Hamilton pipetting robots [19]. They offer greater sensitivity (detection limit of 10^2^–10^3^ DNA copies per reaction vs 10³–10^4^ DNA copies per millilitre), with a shorter time to result (~6 h vs 48 h). This complementary approach combines the broad detection spectrum of 16S rRNA PCR, which can identify other *Neoehrlichia* species and members of the *Ehrlichia* genus, with the advantages of species-specific quantitative PCR, namely faster turnaround times through avoidance of sequencing, higher sensitivity and the ability to monitor bacterial load during treatment. The lack of a commercial serological assay limits diagnosis and epidemiological data. Immunofluorescence using whole bacteria as antigen may be used with an empirical cut-off of 1/32 for IgM and 1/64 for IgG by analogy with rickettsial and chlamydial serology. However, access to molecular testing and immunofluorescence remains limited. This highlights the importance of identifying nearby reference centres providing such molecular testing.

In the absence of guidelines, treatment consists of doxycycline 100 mg bid for 21 days, remaining an effective treatment, although shorter courses might cause relapse [8]. Given the potentially prolonged delays before reporting results (7–29 days in our case), empirical doxycycline therapy may be considered while awaiting microbiological confirmation, particularly in urgent clinical situations and when pre-test probability is high.

## Conclusions

The increasing use of B-cell-depleting therapies combined with the rising prevalence of ticks and *N. mikurensis* infections raises concerns about future trends, particularly given potential severe complications. Despite available PCR tests and effective treatment, low awareness among clinicians contributes to significant diagnostic delays. Future priorities should be given to developing immunofluorescence- and ELISA-based assays to allow for large seroprevalence surveys and to combine molecular- and serological-based diagnosis. Greater clinical recognition of *N. mikurensis* as a cause of persisting febrile illness in immunocompromised patients is warranted.

## Abbreviations

FUO: fever of unknown origin
